# Assessment of Sex-Specific Toxicity and Physiological Responses to Thymol in a Common Bean Pest *Acanthoscelides obtectus* Say

**DOI:** 10.3389/fphys.2022.842314

**Published:** 2022-02-17

**Authors:** Jelica Lazarević, Stojan Jevremović, Igor Kostić, Ana Vuleta, Sanja Manitašević Jovanović, Miroslav Kostić, Darka Šešlija Jovanović

**Affiliations:** ^1^Institute for Biological Research “Siniša Stanković” - National Institute of Republic of Serbia, University of Belgrade, Belgrade, Serbia; ^2^Bayer d.o.o., Belgrade, Serbia; ^3^Institute for Multidisciplinary Research, University of Belgrade, Belgrade, Serbia; ^4^Institute for Medicinal Plants Research “Dr. Josif Pančić”, Belgrade, Serbia

**Keywords:** *Acanthoscelides obtectus*, seed protection, thymol, insecticidal activity, antioxidative defense, detoxification, sexual dimorphism

## Abstract

*Acanthoscelides obtectus* Say (Coleoptera: Chrysomelidae: Bruchinae), is one of the most important pests of the common bean *Phaseolus vulgaris* L. Without appropriate management it may cause significant seed loss in storages. In search for means of environmentally safe and effective protection of beans we assessed biological activity of thymol, an oxygenated monoterpene present in essential oils of many aromatic plants. We studied contact toxicity of thymol on bean seeds and its effects on adult longevity and emergence in F1 generation. Furthermore, we determined acetylcholinesterase (AChE), superoxide dismutase (SOD), catalase (CAT), mixed-function oxidase (MFO), carboxylesterases (CarE) and glutathione S-transferase (GST) activities in response to 24 h exposure of beetles to sublethal and lethal thymol concentrations. Our results showed that thymol decreased adult survival, longevity and percentage of adult emergence. Higher median lethal concentration (LC_50_) was recorded in females indicating their higher tolerance comparing to males. Overall, activities of SOD, CAT and CarE increased at sublethal and MFO increased at both sublethal and lethal thymol concentrations. On the other hand, GST and AChE activities decreased along with the increase in thymol concentrations from sublethal (1/5 of LC_50_, 1/2 of LC_50_) to lethal (LC_50_). Enzyme responses to the presence of thymol on bean seed were sex-specific. In the control group females had lower CarE and higher SOD, CAT and GST activity than males. In treatment groups, females had much higher CAT activity and much lower CarE activity than males. Our results contribute to deeper understanding of physiological mechanisms underlying thymol toxicity and tolerance which should be taken into account in future formulation of a thymol-based insecticide.

## Introduction

The bean weevil *Acanthoscelides obtectus* Say (Coleoptera: Chrysomelidae: Bruchine) is an economically important pest of leguminous crops. Beside the primary host common bean *Phaseolus vulgaris* L. it can also feed on other crops belonging to 11 different genera (Johnson, 1981; Labeyrie, 1990). In a study of Szentesi (2021) 18 legume species are shown to be acceptable, and nine of them support complete development to adults even if seed coat was intact. *A. obtectus* originates from South America but widened its areal of distribution to Europe, North America, Australia and Africa due to human-mediated migrations and tolerance to broad range of environmental conditions (Alvarez et al., 2005). Bean infestation starts in fields by female oviposition into pods and then spreads in storages causing rapid destruction of bean seeds in subsequent generations (Schmale et al., 2002). Larvae feed inside the seeds leading to changes in their mass and nutritional quality (Keszthelyi et al., 2018). Quantitative post-harvest losses in storages due to insect infestation may reach value of 30% in developing countries (Nayak and Daglish, 2018).

Chemical fumigants and contact insecticides are still the main method for storage seed protection (Obeng-Ofori, 2010). However, environmental pollution and threats to human health due to insecticide residues as well as the risk of pest resistance evolution (Guedes et al., 2017; Dar et al., 2020) forced searching for alternative management tools (reviewed in Mohapatra et al., 2015; Daglish et al., 2018; Rajendran, 2020). For example, recent studies on *A. obtectus* have evaluated efficacy of hermetic storage (Freitas et al., 2016), inert dusts (Floros et al., 2018; Lazarević et al., 2018; Prasantha et al., 2019), predators and parasitoids (Iturralde-García et al., 2020), insecticidal products of entomopatogenic bacteria and fungi (Rodríguez-González et al., 2018, 2020), as well as plant-derived products (Kısa et al., 2018; Jevremović et al., 2019; Hategekimana and Erler, 2020; Lazarević et al., 2020).

Among plant-derived products, essential oils (EOs) and their compounds terpenoids and phenylpropanoids exhibit various biological activities against stored product insects including toxicity and sublethal effects on behavior and physiology (reviewed in Nerio et al., 2010; Zibaee, 2011; Kim S. I. et al., 2012; Ebadollahi and Sendi, 2015; Chaudhari et al., 2021). The complex nature of essential oils and artificial blends of their compounds may slow down evolution of pest resistance, whereas low persistence of the volatiles minimizes harmful impact on the environment (Pavela, 2016; Isman, 2020). Additionally, these natural products may contribute to sustainable plant protection through synergy with chemical insecticides (Norris et al., 2018; Reynoso et al., 2018; Ruttanaphan et al., 2019).

Physiological mechanisms of EOs and EOs compounds activity against insects involve neurotoxic interference on cholinergic, GABA-ergic and octopamine pathways (Jankowska et al., 2018), and metabolic reorganization mostly at the level of xenobiotic detoxification, mitochondrial function and antioxidative defense (Liao et al., 2016; Huang et al., 2018; Gao et al., 2020). The activity of acetylcholinesterase (AChE), the enzyme which degrades neurotransmitter acethylcholine, can be inhibited by many terpenoids (López and Pascual-Villalobos, 2010, 2015; Herrera et al., 2015; Al-Nagar et al., 2020; Liu et al., 2021). EOs and EOs compounds may also decrease the activity of enzymes in the mitochondrial electron transport chain, which further provoke increase in free radicals and oxidative damage to macromolecules (Pinho et al., 2014; da Cunha et al., 2015; Kiran et al., 2017; Liao et al., 2018). To defend from oxidative stress, insects induce various enzymatic and non-enzymatic antioxidants (da Cunha et al., 2015; Kiran and Prakash, 2015a,b; Agliassa and Maffei, 2018; Chen et al., 2021). For example, dietary α-pinene, trans-anethole and thymol elevate activities of superoxide dismutase (SOD), catalase (CAT) and glutathione S-transferase (GST) in *Ephestia kuehniella* Zeller larvae (Shahriari et al., 2018). SOD catalyzes the conversion of superoxide anion radical (O_2_^–^) into oxygen (O_2_) and hydrogen peroxide (H_2_O_2_) after which CAT decomposes H_2_O_2_ to water and O_2_. GST metabolizes lipid peroxides and as a major phase II detoxification enzyme catalyzes conjugation of electrophilic xenobiotics with low-molecular antioxidant glutathione. Formed conjugates are less toxic and more water soluble which facilitates their excretion. Mixed-function oxidase (MFO) and carboxylesterase (CarE), phase I detoxification enzymes involved in decomposition of exogenous toxins, can be also induced in the presence of terpenoids (Yotavong et al., 2015; Vasantha-Srinivasan et al., 2018; Gao et al., 2020; Piri et al., 2020; Subaharan et al., 2021). However, inhibition of detoxification enzymes by EOs and EOs compounds has also been reported in insects (Liao et al., 2017; Tak et al., 2017; de Souza et al., 2019; Hu et al., 2019; Shang et al., 2019; Chen et al., 2021; Gaire et al., 2021).

The present study evaluates insecticidal potential of thymol, a natural monoterpenoid phenol, against *A. obtectus*. Thymol (2-isopropyl-5-methylphenol) is the major ingredient of essential oils extracted from aromatic plants belonging to families of Lamiaceae, Apiaceae, Verbenaceae, Asteraceae, Ranunculaceae, Scrophulariaceae and Saururaceae (Escobar et al., 2020). Many of these plants are used as seasonings in human nutrition and as medicinal herbs with anti-inflammatory, analgesic, antimicrobial, antioxidant and other properties (Peter and Shylaja, 2012; Mancini et al., 2015). FEMA expert panel included thymol and thymol containing essential oils in a list of “generally recognized as safe” (GRAS) natural flavors (Cohen et al., 2021). These compounds also show low toxicity to non-target organisms (Charpentier et al., 2014; Yotavong et al., 2015; Pavela et al., 2020) and various adverse effects on fitness of pest insects (Abdelgaleil et al., 2021b).

In *A. obtectus* thymol applied as fumigant induced high mortality and decreased adult longevity, fecundity, penetration of larvae into bean seeds and adult emergence (Regnault-Roger and Hamraoui, 1995). Our study was aimed to determine residual contact toxicity of thymol by monitoring adult survival and progeny production and to explore the physiological basis of thymol toxicity by measuring activities of AChE, SOD, CAT, MFO, CarE, and GST. Additionally, since females and males of this species differently responded to various chemical stressors (Papachristos and Stamopoulos, 2002; Papachristos et al., 2004; Lazarević et al., 2013, 2018, 2020; Šešlija Jovanović et al., 2014) we assessed if changes in survival and enzyme activities were sex-specific.

## Materials and Methods

### Insects and Rearing Conditions

*Acanthoscelides obtectus* used in this study originated from the laboratory population maintained on the common bean (*Phaseolus vulgaris* c.v. “gradištanac”) seeds for more than 250 generations. During the experiment, beetles were kept in heating incubators at 27 ± 1°C, 12 h:12 h light:dark photoperiod and 55 ± 10% relative humidity. Bean seeds were chemically untreated and frozen prior to usage to avoid any possible infestation with external pests.

### Residual Contact Toxicity of Thymol on Bean Seeds

Thymol purchased from Sigma-Aldrich (cat. no. W306606) was dissolved in acetone. Bean seeds (10 g) were put in 90 mL glass jars and treated with 300 μL of either thymol solutions or solvent (control). Five thymol concentrations were applied for females (60, 90, 105, 120, and 150 mg/kg of beans) and males (30, 45, 60, 75, and 90 mg/kg of beans). Treated seeds were mixed manually for 5 min and left in open jars for 20 min to evaporate the solvent. Then, 10 adult females or males (one day old) were introduced into the jar, covered with a piece of cloth fixed with rubber. Eight replicates per sex per thymol concentration and control (acetone) were analyzed. The number of dead insects was estimated daily until all insects died. Percentage of dead insects after 24 h of treatment was used to determine lethal thymol concentrations. Beetle longevity and age-specific mortality were also observed.

### Thymol Effects on F1 Progeny Production

Bean seeds (20 g) were put in 200 mL glass jars and treated with 600 μL of either thymol solution or solvent (control). Applied thymol concentrations were 30, 45, 60, 75, 90, 105, and 120 mg/kg of beans. After 5 min of treated seed mixing and 20 min of solvent evaporation, five pairs of one day old bean weevils, i.e., five females and five males, were introduced into each jar, covered with a piece of cloth fixed with rubber and kept in heating incubators until the emergence of the progeny. Emerged adults were counted daily until the end of emergence. Total number of emerged insects as well as the number of emerged females and males were used to determine the inhibition rate (IR%) of emergence according to the formula:


$$I\,R\%=\frac{N\,c-N\,t}{N\,c}\times 100$$

where N_*c*_ and N_*t*_ were total numbers of emerged adults in control and treatment jars, respectively.

### Enzyme Assays

Activities of enzymes (AChE, SOD, CAT, MFO, CarE, GST) were determined in female and male beetles exposed to sublethal (1/5 of LC_50_, 1/2 of LC_50_) and lethal concentrations (LC_50_) of thymol for 24 h. The enzyme extracts were prepared by pulverization of batches of 20 frozen beetles under liquid nitrogen in a mortar with the pestle. After the addition of cold 50 mM K-phosphate buffer pH 7.4 containing 1 mM EDTA and 1 mM PMSF (1:10 tissue to buffer ratio), homogenates were sonicated (2 × 15 s) and centrifuged at 4°C, 16,000 *× g* for 30 min. The supernatants were collected and used for the determination of enzymes activities and total protein content. All enzyme assays were performed at 30°C. The total protein content was quantified according to Bradford (1976) with bovine serum albumin (BSA) as the standard and enzyme activities were expressed in units (U) per mg of proteins.

The activity of AChE was determined according to the method of Ellman et al. (1961). During the reaction, thiol groups released from substrate acetyl-thiocholine iodide (ACTH) bind to 5,5′-dithio-bis(2-nitrobenzoic acid) (DTNB) and form yellow 5-thio-2-nitrobenzene (TNB). The reaction was carried out in 50 mM phosphate buffer pH 7.9 and the change in absorbance was monitored at 406 nm (ε = 13,330 M^–1^ cm^–1^). One enzyme unit (U) was defined as the amount of enzyme that forms 1 nmol TNB per min.

The activity of SOD was assayed by the method of Misra and Fridovich (1972), which is based on the capacity of SOD to inhibit autoxidation of adrenaline to adrenochrome at pH 10.2 (50 mM sodium carbonate buffer). The change in absorbance was monitored at 480 nm. One unit of SOD activity was defined as the amount of enzyme causing 50 % inhibition of the adrenaline autoxidation.

CAT activity was determined by the method of Claiborne (1984). The rate of hydrogen peroxide (H_2_O_2_) decomposition in 50 mM phosphate buffer pH 7.0 was determined according to the change in absorbance at 240 nm (ε = 43.6 M^–1^ cm^–1^). One unit of CAT activity was defined as the amount of enzyme that catalyzed the decomposition of 1 μmol of H_2_O_2_ per min.

The activity of MFO was quantified indirectly by the heme peroxidation method (Brogdon et al., 1997). TMBZ (3, 3′, 5, 5′-tetra-methylbenzidine) dissolved in methanol and sodium acetate buffer pH 5.0 was used as a hydrogen donor substrate. The reaction started with adding a drop of hydrogen peroxide. After 5 minutes of incubation absorbance was read at 630 nm. Cytochrome c was used as an internal standard and the enzyme unit was expressed as pmol of cytochrome c equivalents per min.

CarE activity was determined by the method of Wu et al. (1998) by using p-nitrophenyl acetate (p-NA) as a substrate. The enzyme hydrolyzes acetate ester and forms p-nitrophenol (p-NP) which absorbs at 405 nm (ε = 12,800 M^–1^ cm^–1^). The enzyme unit was defined as the amount of enzyme which generates 1 nmol of p-NP per min.

The activity of GST was determined by the method of Habig et al. (1974). The method is based on the reaction of CDNB with the SH group of GSH which was performed in 100 mM potassium phosphate buffer pH 6.5. The change in absorbance was measured at 340 nm (ε = 9,600 M^–1^ cm^–1^) and the enzyme unit was defined as the amount of enzyme that generate 1 nmol of CDNB-GSH conjugate per min.

### Statistical Methods

Lethal thymol concentrations after 24 h of exposure were estimated by probit analysis (Finney, 1971) and their values were compared between females and males according to overlapping confidence intervals. Based on mortality data during the beetles life time Kaplan-Meier survival probability was calculated, survival analysis was performed and survival distribution was compared among thymol concentrations by log-rank test. Parameters *a* (initial mortality) and *b* (exponential increase in mortality over time) of the Gompertz model (instantaneous mortality at age *x* = *a*×*e^bx^*) were determined by using WinModest software and compared between control and thymol treated beetles by using the log-likelihood-ratio test (Pletcher, 1999). Also, Gompertz parameters were compared between females and males of the control group and group treated with 60 mg of thymol/kg of beans.

Kolmogorov-Smirnov test of normality and Bartlett’s test for homogeneity of variances were applied on data transformed in order to achieve assumptions for parametric ANOVA. Arcus sinus square root transformation was used for the percentage of 24 h adult mortality and the percentage of adult emergence inhibition. Data on the number of emerged adults and adult longevity were square root transformed. Assumption of normality of distribution was violated for square root transformed female and male longevities. Accordingly, to assess the impact of thymol concentration on adult longevity we used non-parametric Kruskal-Wallis ANOVA and Dunn’s *post-hoc* test, whereas 24 h mortality and adult emergence were analyzed by parametric 1-way ANOVA and Duncan’s *post-hoc* test. To reveal the significance of the differences in the number of emerged females and males we performed 1-way repeated measures ANOVA with sex as within-subject factor and thymol concentration as between-subject factor.

Enzyme activities were analyzed by 2-way ANOVA with thymol concentration and sex as fixed factors. Carboxylesterase activity was log-transformed whereas untransformed data on other enzyme activities satisfied parametric ANOVA assumptions. A posteriori comparisons (least square means contrasts) were applied to assess the significance of enzyme activity differences between sexes within each thymol concentration. Also, 1-way ANOVAs followed by Duncan’s *post-hoc* test were carried out to reveal the significance of thymol concentration effects on enzyme activities separately in females and males. All analyses were carried out with the software Statistica 7.0 (StatSoft, Inc., Tulsa, OK, United States).

## Results

### Acute Thymol Toxicity Against *Acanthoscelides obtectus*

**FIGURE 1:**
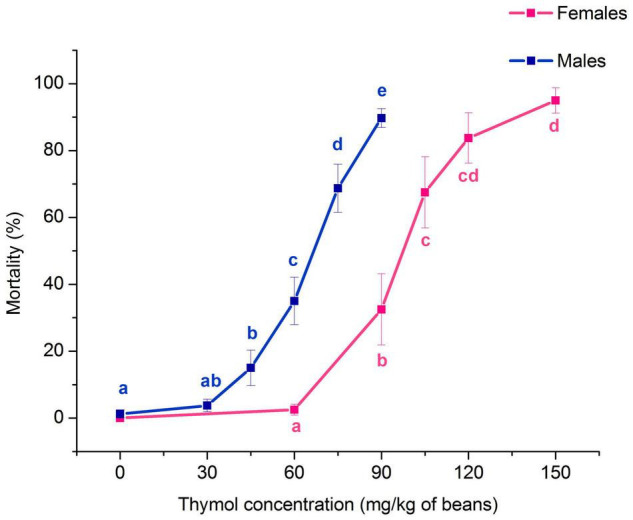
Impact of different concentrations of thymol on *Acanthoscelides obtectus* mortality after 24 h of exposure to thymol treated bean seeds (means ± SE for 8 replicates). Significant differences among experimental groups within each sex are marked with different letters (a – e) (Duncan’s *post-hoc* test, *p* < 0.05).

Thymol concentration significantly affected the percentage of *A. obtectus* mortality both in females (*F*_4,35_ = 24.81, *p* < 0.001) and males (*F*_5,42_ = 43.17, *p* < 0.001). Mortality of females and males was significantly increased at concentrations equal or higher than 90 and 45 mg/kg of beans, respectively (Figure 1). Concentration-mortality response fitted the probit distribution (Pearson’s test in Table 1). Higher resistance of females than males to thymol was confirmed by higher low lethal (LC_30_) and lethal concentrations (LC_50_, LC_99_) with non-overlapping confidence intervals.

**TABLE 1:** Residual contact toxicity of thymol against adult females and males of *Acanthoscelides obtectus*.

	Slope (CI)	LC_30_ (CI)	LC_50_ (CI)	LC_99_ (CI)	χ^2^	*p*
Females	8.73 ± 0.79 (7.17, 10.28)	85.8 (80.7, 89.7)	98.4 (94.2, 102.3)	181.8 (165.6, 207.0)	3.84	0.279
Males	8.68 ± 0.80 (7.11, 10.25)	55.2 (51.3, 58.5)	66.0 (62.7, 69.0)	108.9 (100.8, 120.9)	1.02	0.795

*LC – lethal thymol concentrations expressed in mg/kg of beans leading to 30, 50 and 99% beetle mortality (LC_30_, LC_50_, and LC_99_, respectively); CI – 95% confidence interval; χ^2^ and P – Pearson’s goodness-of-fit test (df = 3).*

### *Acanthoscelides obtectus* Longevity and Time-Mortality Responses to Thymol

**FIGURE 2:**
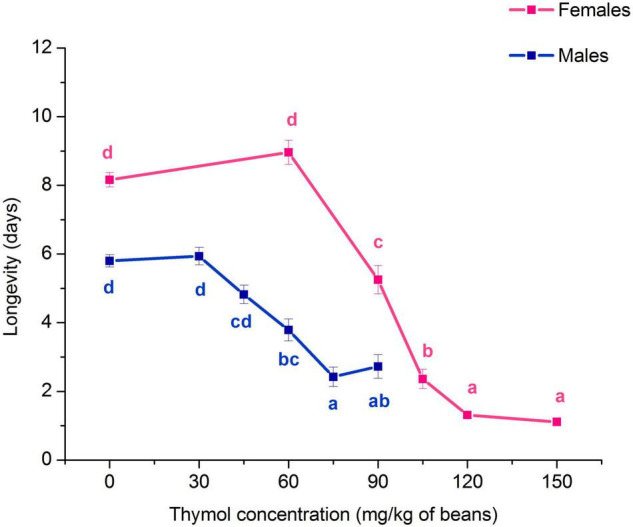
Changes in longevity of *Acanthoscelides obtectus* females and males (means ± SE for 8 replicates) in response to chronic exposure to thymol treated bean seeds. Significant differences among experimental groups within each sex are marked with different letters (a - d) (Dunn’s *post-hoc* test, *p* < 0.05).

Exposure to thymol negatively affected *A. obtectus* adult longevity. Both females and males lived shorter comparing to control beetles (Kruskal-Wallis ANOVA, females: *H*_4,399_ = 227.68, *p* < 0.001; males: *H*_5,479_ = 125.48, *p* < 0.001). Significant longevity decrease can be observed at concentrations ≥ 90 mg/kg of beans in females and 60 mg/kg of beans in males (Figure 2).

**FIGURE 3:**
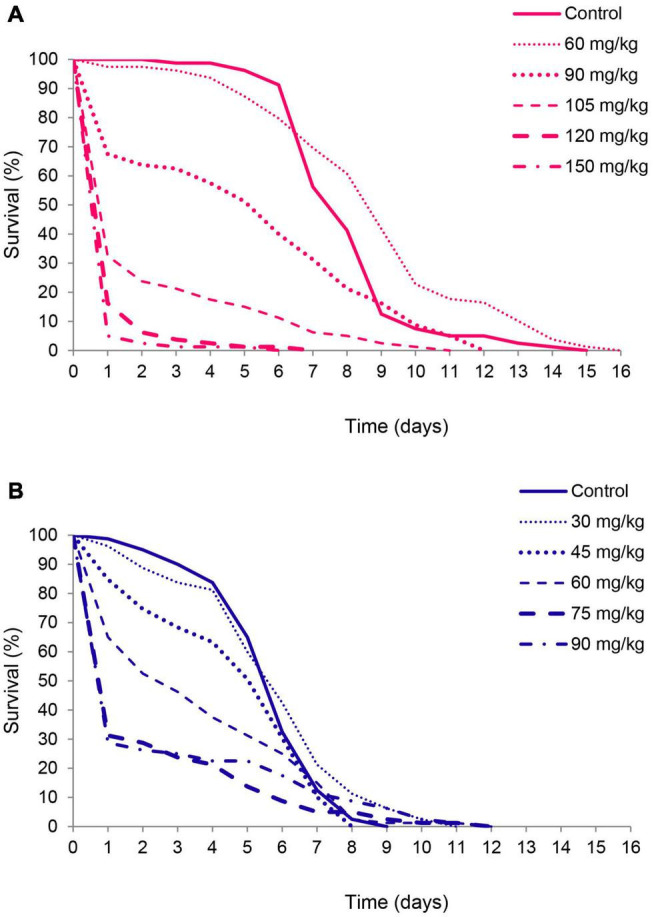
Survival curves for female **(A)** and male *Acanthoscelides obtectus* **(B)** exposed to different thymol concentrations.

As revealed by Kaplan-Meier analysis thymol applied on bean seeds also affected survival distribution over time in females (χ^2^ = 368.12, df = 5, *p* < 0.001) and males (χ^2^ = 58.35, df = 5, *p* < 0.001). Beetles from the treatment groups started to die earlier than control beetles (Figure 3) and had higher initial mortality at thymol concentrations ≥ 90 mg/kg of beans in females and 30 mg/kg of beans in males (Gompertz parameter *a* in Table 2). This higher initial mortality was related to slower mortality increase with advanced age and higher maximum longevity (Figure 3, Gompertz parameter *b* in Table 2). In the control group males lived shorter due to accelerated aging rate, whereas in beetles exposed to 60 mg of thymol / kg of beans shorter life of males was a consequence of much higher initial mortality (higher parameter *a*) which could not be compensated by retarded aging (lower parameter *b*) (Table 2).

**TABLE 2:** Gompertz mortality parameters (*a* – initial mortality; *b* – exponential increase in mortality with age) and 95% confidence intervals (CI) in *Acanthoscelides obtectus* females and males exposed to different thymol concentrations.

	Gompertz mortality parameters			
Concentration (mg/kg of beans)	*a* (× 10^–2^)	(CI)	*b*	(CI)
**Females**				
0	1.09	(0.61, 1.95)	0.40	(0.34, 0.47)
60	1.35	(0.74, 2.46)	0.30*	(0.25, 0.37)
90	9.48*	(6.08, 14.77)	0.15*	(0.10, 0.25)
105	61.63*	(46.24, 82.14)	0.19*	(0.09, 0.41)

**Males**				
0	0.72	(0.33, 1.57)	0.70*^C^*	(0.58, 0.84)
30	2.49*	(1.41, 4.40)	0.41*	(0.33, 0.51)
45	3.60*	(1.96, 6.61)	0.45*	(0.34, 0.58)
60	16.47^*,*C*^	(11.01, 24.66)	0.14^*,*B*^	(0.08, 0.26)

*Significant differences from the control group are marked with asterisks (log-likelihood ratio test, df = 1, p < 0.05). Significant differences between females and males of control and 60 mg of thymol / kg of beans treatment group are marked with B (p < 0.01) and C (p < 0.001).*

### Thymol Impact on *Acanthoscelides obtectus* Adult Emergence

Results presented in Table 3 show that the number of emerged adults decreased significantly at concentrations ≥ 60 mg/kg of beans (female emergence: *F*_5,42_ = 21.12, *p* < 0.001; male emergence: *F*_5,42_ = 21.14, *p* < 0.001; total emergence: *F*_5,42_ = 22.19, *p* < 0.001). Similarly, the percentage of emergence inhibition was significantly affected by thymol concentration (females: *F*_4,35_ = 19.51, *p* < 0.001; males: *F*_*4,35*_ = 16.81, *p* < 0.001; females+males: *F*_*4,35*_ = 18.80, *p* < 0.001). The concentration that provoked 50% emergence inhibition was estimated to be 57.3 mg/kg of beans (CI = 0.179; 0.201). Significant influence of thymol concentration on adult emergence and emergence inhibition was also confirmed by repeated measures ANOVA (*F*_5,42_ = 22.32, *p* < 0.001 and *F*_4,35_ = 19.05, *p* < 0.001, respectively). On average, more males than females emerged in F1 generation (within-subject sex: *F*_1,42_ = 8.20, *p* = 0.007) but emergence inhibition was not sex specific (*F*_1,35_ = 0.04, *p* = 0.846). Additionally, slope of thymol concentration – F1 progeny number and thymol concentration – emergence inhibition response did not differ between females and males (concentration × sex interaction: *F*_5,42_ = 0.19, *p* = 0.964 and *F*_*4,35*_ = 0.50, *p* = 0.734, respectively).

**TABLE 3:** Adult emergence in F1 generation and emergence inhibition (means ± SE for 8 replicates) in *Acanthoscelides obtectus* depending on thymol concentration (Conc) in parental generation.

Thymol	Number of emerged adults			Emergence inhibition (%)		
Conc (mg/kg of beans)	Females	Males	Total	Females	Males	Total
0	43.5 ± 6.6^c^	47.5 ± 5.2^c^	91.0 ± 11.3^c^			
30	48.4 ± 3.4^c^	55.1 ± 6.3^c^	103.5 ± 9.3^c^	−11.2 ± 7.8^a^	−16.1 ± 13.3^a^	−13.7 ± 10.2^a^
45	33.9 ± 4.6^c^	35.5 ± 4.0^c^	69.4 ± 8.3^c^	22.1 ± 10.5^b^	25.3 ± 8.4^b^	23.8 ± 9.1^b^
60	18.6 ± 5.9^b^	19.5 ± 5.8^b^	38.1 ± 11.6^b^	57.2 ± 13.6^c^	58.9 ± 12.1^bc^	58.1± 12.7^*b*^
75	5.8 ± 2.9^a^	8.9 ± 4.1^a^	14.6 ± 6.7^a^	86.8 ± 6.7^d^	81.3 ± 8.7^cd^	83.9 ± 7.4^c^
90	3.4 ± 1.9^a^	3.9 ± 1.8^a^	7.3 ± 3.7^a^	92.2 ± 4.4^d^	91.8 ± 3.9^d^	92.0 ± 4.0^c^

*Values marked with different letters (a, b, c, d) within columns indicate significant differences among treatments (Duncan’s post-hoc test, p < 0.05).*

### Enzyme Activities in *Acanthoscelides obtectus* Exposed to Sublethal and Lethal Thymol Concentrations

**FIGURE 4:**
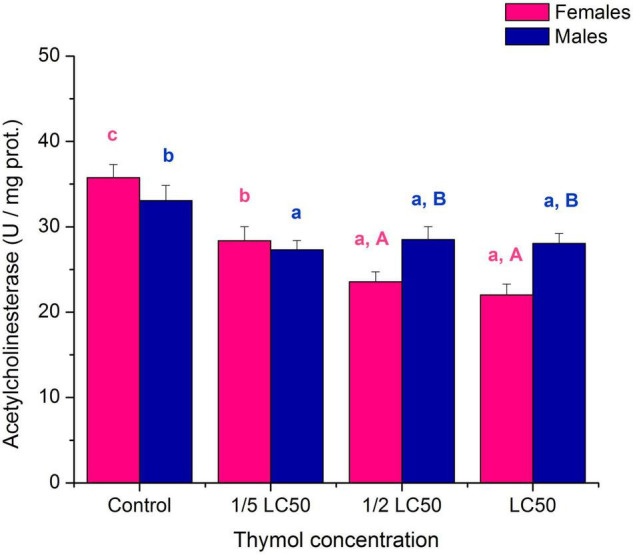
Activity of acetylcholinesterase (mean ± SE for 5 replicates) in females and males of *Acanthoscelides obtectus* exposed to different thymol concentrations. Different lowercase letters (a, b, c) mark significant differences among concentrations within each sex (Duncan’s *post-hoc* test, *p* < 0.05), whereas different uppercase letters (A, B) show significant differences between females and males within each thymol concentration (LSM contrasts, *p* < 0.05).

The neurotoxic effect of thymol on *A. obtectus* adults was revealed by inhibition of AChE activity both at sublethal and lethal concentrations (Figure 4; significant “concentration” term in Table 4). The slope of AChE inhibition differed between females and males (significant “sex × concentration” term in Table 4). Influence of thymol concentration was highly significant in females (*F*_3,16_ = 18.77, *p* < 0.001), and males (F_3,16_ = 3.40, *p* = 0.044). At median lethal concentration LC_50_ AchE was inhibited about 38% in females and 15% in males (Figure 4).

**TABLE 4:** F and *p* values from 2-way ANOVA testing significance of main and interaction effects of sex and thymol concentration on activities of acetylcholinesterase (AChE), superoxide dismutase (SOD), catalase (CAT), mixed-function oxidase (MFO), carboxylesterase (CarE), and glutathione S-transferase (GST) in *Acanthoscelides obtectus*.

Source of	AChE		SOD		CAT		MFO		CarE		GST	
variation	F	*p*	F	*p*	F	*p*	F	*p*	F	*p*	F	*p*
Sex (df: 1, 32)	3.2	0.082	0.0	0.935	2366.0	**<0.001**	31.4	**<0.001**	682.7	**<0.001**	22.5	**<0.001**
Concentration (df: 3, 32)	17.6	**<0.001**	9.7	**<0.001**	19.8	**<0.001**	41.6	**<0.001**	21.3	**<0.001**	150.1	**<0.001**
Sex × Concentration (df: 3, 32)	4.6	**0.009**	8.6	**<0.001**	1.2	0.329	3.4	**0.029**	7.0	**<0.001**	43.6	**<0.001**

*Significant effects are marked in bold.*

**FIGURE 5:**
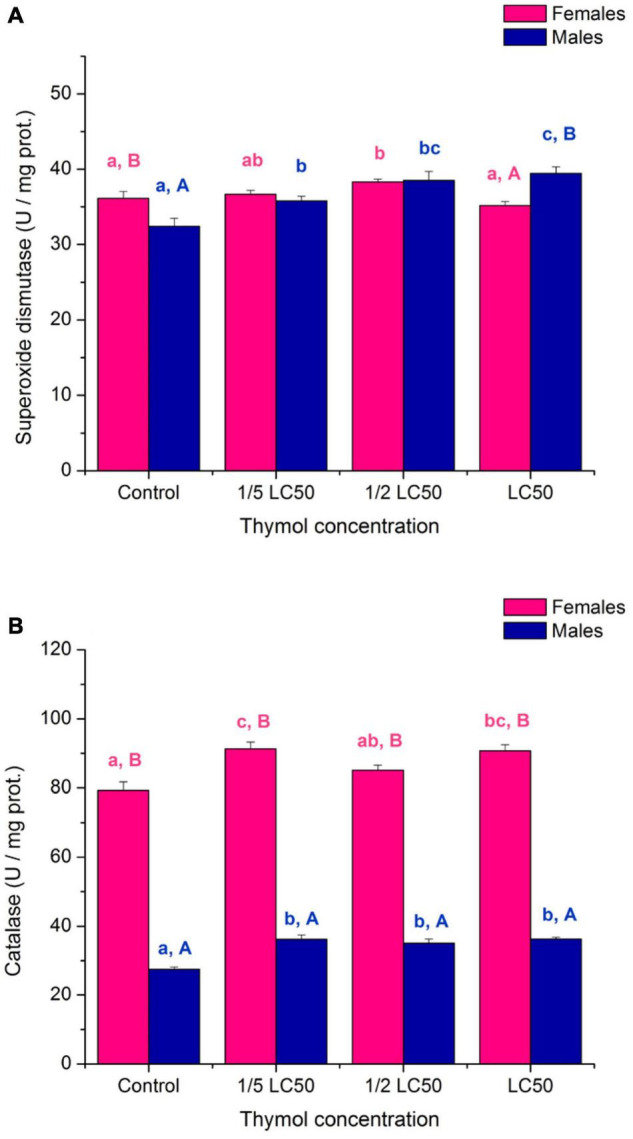
Activity of superoxide dismutase **(A)** and catalase **(B)** in females and males of *Acanthoscelides obtectus* exposed to different thymol concentrations (mean ± SE for 5 replicates). Different lowercase letters (a, b, c) mark significant differences among concentrations within each sex (Duncan’s *post-hoc* test, *p* < 0.05), whereas different uppercase letters (A, B) show significant differences between females and males within each thymol concentration (LSM contrasts, *p* < 0.01).

On average, activities of antioxidative enzymes SOD and CAT were elevated in the presence of thymol (Figure 5; significant “concentration” term in Table 4). Shape of thymol concentration – activity response depended on sex (significant “sex × concentration” terms in Table 4) although both females (SOD: *F*_3,16_ = 4.6, *p* = 0.017; CAT: *F*_3,16_ = 8.35, *p* = 0.001) and males (SOD: F_3,16_ = 10.97, *p* < 0.001; CAT: *F*_3,16_ = 19.47, *p* < 0.001) were significantly affected by thymol concentration. Activity of catalase was about 2.5 times higher in females than males across all examined concentrations. In contrast, differences in SOD activity were recorded only in control (higher activity in females) and LC_50_ group (higher activity in males) (Figures 5A,B).

**FIGURE 6:**
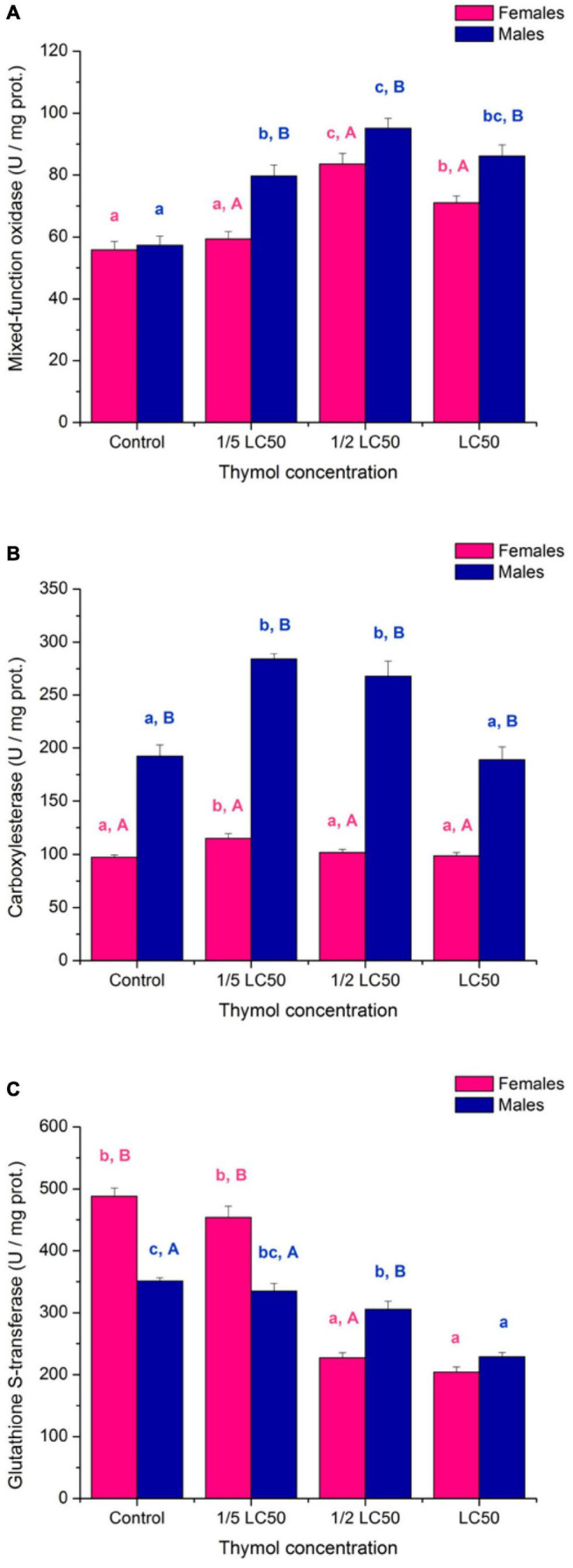
Activity of mixed-function oxidases **(A)**, carboxylesterase **(B)** and glutathione S-transferase **(C)** in females and males of *Acanthoscelides obtectus* exposed to different thymol concentrations (means ± SE for 5 replicates). Different lowercase letters (a, b, c) mark significant differences among concentrations within each sex (Duncan’s *post-hoc* test, *p* < 0.05), whereas different uppercase letters (A, B) show significant differences between females and males within each thymol concentration (LSM contrasts, *p* < 0.05).

Activity of mixed-function oxidases was elevated in treated beetles (Figure 6A; significant “concentration” term in Table 4). Both females (*F*_3,16_ = 21.93, *p* < 0.001) and males (*F*_3,16_ = 22.88, *p* < 0.001) were significantly affected by thymol concentration but female MFO was less sensitive (Figure 6A; significant “sex × concentration” term in Table 4). Males exposed to thymol had higher MFO activity than females (Figure 6A; significant “sex” term in Table 4).

Carboxylesterase activity was induced at sublethal thymol concentrations (Figure 6B; significant “concentration” term in Table 4). Significant thymol influence was detected both in females (*F*_3,16_ = 5.38, *p* = 0.009) and males (*F*_3,16_ = 17.49, *p* < 0.001) but induction was more expressed in males than females (Figure 6B; significant “sex × concentration” term in Table 4). At all examined concentrations CarE activity was higher in males than females (Figure 6B; significant “sex” term in Table 4).

Inhibition of glutathione S-transferase activity by thymol is another possible mechanism of its toxicity (Figure 6C; significant “concentration” term in Table 4). Both females (*F*_3,16_ = 136.13, *p* < 0.001) and males (*F*_3,16_ = 30.73, *p* < 0.001) were significantly affected by thymol concentration but inhibition was steeper in females than males (Figure 6C; significant “sex × concentration” term in Table 4). Control and 1/5 of LC_50_ females had about 40% higher GST activity than males, whereas no difference could be recorded at LC_50_ (Figure 6C).

## Discussion

### Adverse Effects of Thymol on Adult Fitness Traits

Similar to the results on residual contact toxicity of thyme EO against the bean weevil (Lazarević et al., 2020) we found that its major compound thymol also significantly affected 24 h mortality and longevity of females and males, and progeny production in F1 generation. The ratio of median lethal concentrations obtained for thymol and thyme EO (98.4 vs. 255.0 mg/kg of beans in females and 66.0 vs. 172.2 mg/kg of beans in males) corresponds to thymol concentration of 43.52% in thyme EO (Jevremović et al., 2019) and suggests that thymol was the major determinant of bean weevil mortality induced by thyme EO. It appeared that other EO compounds either had negligible impact on the acute toxicity of EO or some compounds contribution counteracted the antagonistic effects of others. In difference to our results studies on fumigant toxicity of thyme EO containing 47.5% thymol (Regnault-Roger et al., 1993) and pure thymol (Regnault-Roger and Hamraoui, 1995) point to the significant contribution of other compounds. Using different bean weevil populations and different modes of botanical application may account for disagreement between results of the studies. Besides, although both EOs were extracted from thyme belonging to thymol chemotype their composition was different. For example, thyme EO from the study of Regnault-Roger and colaborators contained caryophyllene, bicyclic sesquiterpene, which had both fumigant and residual toxic effects on stored product insects (Lee et al., 2008; Sun et al., 2020).

On the other hand, ratio of median effective concentration (EC_50_) for adult emergence inhibition rate on thymol (57.3 mg/kg of beans) and thyme EO (65.7 mg/kg of beans, Lazarević et al., 2020) suggests an important role of other compounds in reducing adult emergence. Since pure thymol seems to affect F1 progeny number mainly through the adverse effect on male survival (similarity of EC_50_ value for adult emergence inhibition to LC_50_ for males on thymol) it is possible that other compounds in thyme EO additionally reduced emergence through adverse effects on mating, fecundity, larval penetration into seeds and/or preadult survival. Even sublethal concentrations of monoterpenes could have a significant impact on fitness traits and behavior of the bean weevil (Regnault-Roger and Hamraoui, 1995; Jevremović et al., 2019; Hategekimana and Erler, 2020) and other pests (Hummelbrunner and Isman, 2001; Wang et al., 2009; Tak and Isman, 2017; de Melo et al., 2018; Abdelgaleil et al., 2021a; Barbosa et al., 2021; de Andrade Brito et al., 2021; Ruiz et al., 2021). Because thymol also affects bean weevil fitness traits (Regnault-Roger and Hamraoui, 1995) and deters oviposition (Jevremović et al., 2019) we suppose that various synergistic and antagonistic interactions of thymol with other monoterpenes might take part in determining the number of emerged adults. In other pest species such interactions can affect repellence, feeding deterrence, female attraction to males, egg viability, adult emergence and locomotion (Singh et al., 2009; Tak and Isman, 2017; Youssefi et al., 2019; Ataide et al., 2020; López et al., 2021).

Insecticidal, repellent, antifeedant, oviposition deterrent and growth reducing effects of thymol have been confirmed in stored products (Kim et al., 2010; Szczepanik et al., 2012; Brari and Thakur, 2015; Oliveira et al., 2017, 2018; Shahriari et al., 2017; Wahba et al., 2018; da Camara et al., 2022), agricultural (Hummelbrunner and Isman, 2001; Wilson and Isman, 2006; Pavela, 2011a; Koul et al., 2013; Lima et al., 2020; Valcárcel et al., 2021) and medically important pest insects (Pavela, 2011b; Zahran and Abdelgaleil, 2011; Govindarajan et al., 2013; Youssefi et al., 2019). Influence of thymol on stored products insects depends on insect species, mode of application and sex. For example, in residual contact assays, *Tribolium castaneum* was more sensitive than *Sitophilus oryzae* (Kanda et al., 2017), whereas in fumigant assays efficacy of thymol was equal in these species (Brari and Thakur, 2015). Relative ranking of terpene efficacy also depended on the mode of application. Comparisons of thymol and linalool revealed similar toxicity and oviposition inhibition effect of thymol against the bean weevil in fumigant assay (Regnault-Roger and Hamraoui, 1995), whereas in residual contact assays thymol was more effective in reducing survival and oviposition (Jevremović et al., 2019). About ten times higher vapor pressure of linalool than thymol (0.157 and 0.016 mmHg, respectively) may account for such a relationship. This result is consistent with the findings of other authors that thymol bioactivity is superior in contact assays (Waliwitiya et al., 2005; Hieu et al., 2014; Wahba et al., 2018).

### Thymol Toxicity Is Sex-Specific

Bean weevil females are more tolerant to monoterpenes and essential oils (Regnault-Roger and Hamraoui, 1995; Papachristos and Stamopoulos, 2002; Papachristos et al., 2004; Lazarević et al., 2020), which is confirmed in our study with residual contact toxicity of thymol where we recorded higher median lethal concentration and lower initial mortality in females than males. In other insect species, females are usually more tolerant to plant-derived compounds (Yeom et al., 2012; Theou et al., 2013; Jang et al., 2017; Park et al., 2017; Pavela et al., 2021). Sex differences depend on applied compounds and the method of application. Jang et al. (2017) detected higher tolerance of female *Drosophila suzuki* to topically applied citronellal, citronellol and isopulegol, but not after fumigant application. Only essential oils rich in α-pinene were more toxic to *Musca domestica* males (Pavela et al., 2021). In the bean weevil, females were about 3.5 times more resistant to α-terpineol and only 1.2 times more resistant to α-pinene (Papachristos et al., 2004). Likewise, bean weevil females were about 5 times more resistant to *Mentha microphylla* EO and 1.5 times more resistant to *Lavandula hybrida* EO (Papachristos and Stamopoulos, 2002) and thymol (present results). Possible explanations of sexual dimorphism in toxicity of plant-derived compounds against bean weevil are differences in body size and cuticle composition (Tucić et al., 1996; Gołebiowski et al., 2008) as well as differences in physiology (Šešlija et al., 1999; Lazarević et al., 2012, 2020; Arnqvist et al., 2017; Zhang et al., 2020) that may affect compound bioavailability and bean weevils’ innate ability to cope with chemical stressors.

Our results showing a lower exponential increase in mortality with age in thymol treatment groups and higher maximum longevity at sublethal thymol concentrations imply that individuals that survived after 24 h of exposure possibly had and/or induced some kind of defense responses. To explore mechanisms of thymol toxicity and tolerance, we determined the activity of six enzymes and found that thymol gradually inhibited activities of AChE and GST, elevated activities of SOD, CAT and CarE at sublethal concentrations and MFO at both sublethal and lethal concentrations. Generally, insects resistant to chemical insecticides and plant-derived compounds have higher activities of AChE, SOD and detoxification enzymes (Attia et al., 2017; Roy and Prasad, 2018; Akami et al., 2019; Senthil-Nathan, 2020). In agreement with this, we found that in the absence of thymol females, the more tolerant sex, contained a higher level of low-molecular thiols (Lazarević et al., 2020) and had higher activities of SOD, CAT and GST (present results).

### Role of Antioxidative Enzymes in Tolerance to Thymol

After exposure to sublethal thymol concentrations, SOD increased both in females and males whereas at lethal concentration it was increased only in males. SOD responds first to oxidative stress induced by xenobiotics and protects cells from dangerous free radicals. Without efficient scavenging of its product of reaction H_2_O_2_ by catalase and other peroxidases, it may damage macromolecules, accelerate aging and reduce insect survival and longevity. For instance, bean weevil females exposed to thyme essential oil had lower a level of damaged lipids (Lazarević et al., 2020). In consistence with our results, SOD and CAT were elevated by thymol in *Ephestia kuehniella* larvae (Shahriari et al., 2018), by carvacrol, p-cymene and γ-terpinene in *S. littoralis* larvae (Agliassa and Maffei, 2018), by carvacrol in *Lymantria dispar* larvae (Chen et al., 2021), by ethanolic extract of *Acalypha wilkesiana* leaves in *Callosobruchus maculatus* adults (Oni et al., 2019) and by *Boswellia carterii* EO in adults of two *Callosobruchus* species (Kiran et al., 2017). SOD and CAT responses to botanicals may vary depending on insect species, duration of exposure, botanical type and concentration. Several studies have shown that more resistant species had higher SOD and CAT activity, higher induction of activity and/or higher CAT to SOD ratio (Kiran et al., 2017; Petrović et al., 2019). Also, in some species, botanicals can reduce SOD and/or CAT activity at high concentrations (Oni et al., 2019; Rajkumar et al., 2019).

Here we recorded higher CAT activity and CAT to SOD activity ratio in females across all examined thymol concentrations that could account for sex-specific differences in tolerance to thymol. Similar result was obtained in *A. obtectus* populations selected for early and late reproduction (Šešlija et al., 1999) indicating that a higher CAT and CAT to SOD ratio is characteristic of this species. Tasaki et al. (2017) suggested that females of eusocial Isoptera and Hymenoptera have high CAT activity whereas in solitary insects CAT activity is lower in females than males. However, there are also examples of higher CAT activity in females of solitary species under control (Sharma et al., 1995) and stressful conditions (Rovenko et al., 2015; Manna et al., 2020; Wang et al., 2020). Several studies have shown that CAT accumulates in insect ovaries providing protection to developing oocytes from oxidative damage (e.g., de Jong et al., 2007; Diaz-Albiter et al., 2011). We speculate that such mechanism might contribute to higher CAT activity in *A. obtectus* females which emerge with about 30 mature chorionated eggs in the lateral oviduct (Leroi, 1981). In the absence of mating antioxidants accumulated in eggs would be fully available for defense against xenobiotics.

### Thymol Inhibits Activities of AChE and GST

Our observation about inhibition of neurotransmitter enzyme AChE and detoxification enzyme GST by thymol in bean weevil females and males agrees with findings of other studies on physiological mechanisms of monoterpenes and essential oils toxicity (Mojarab-Mahboubkar et al., 2015; Liao et al., 2016, 2017; Agliassa and Maffei, 2018; Yang et al., 2018; Chen et al., 2021; Fouad and Abotaleb, 2021). Inhibition of AChE leads to accumulation of acetylcholine at nerve synapses, and thus permanent conduction of nerve impulses, ataxia, convulsions and death (Rattan, 2010). AChE activity was more reduced in females than males so that males had higher activity at thymol concentration 1/2 of LC_50_ and LC_50_. In difference to our results, AChE of female adults of *Blatella germanica* and *Drosophila suzuki* were less sensitive to in vitro inhibition with thymol (Yeom et al., 2012; Park et al., 2016). The discrepancy between toxicity and AChE inhibition by botanical insecticides suggests differences in other neurological or enzymatic target sites. A weak correlation has been found between AChE activity and insecticide resistance in *D. melanogaster* (Charpentier and Fournier, 2001). Therefore, the relationship between AChE activity and resistance to chemical stress may depend on insect species and population or applied compound. For example, although more resistant to fumigation with lemongrass essential oil AChE activity in treated females of *Callosobrucus maculatus* was lower than in males (de Souza et al., 2019). To fully understand AChE-thymol resistance relationship further researches are needed to reveal how thymol affects the level of cholinergic and non-cholinergic AChE isoforms. Non-cholinergic AChE is important for insect fecundity and defense against xenobiotics, and, compared to cholinergic isoform, exhibits lower catalytic efficiency and higher resistance to inhibiton by insecticides (Kim Y. H. et al., 2012; Kim et al., 2014; Lu et al., 2012; Hwang et al., 2014; Lee et al., 2015; Kim and Lee, 2018). Therefore, a higher level of protective non-cholinergic isoform might provide higher thymol resistance to females despite the lower activity. Several studies have shown sex-dependant relative expression of genes encoding the two isoforms (Zhao et al., 2013; Salim et al., 2017).

Inhibition of GST by thymol is an important feature from the pest management point of view because it could interfere with the detoxification in insects and lead to enhanced activity of conventional insecticides (Ismail, 2021). In *Trichoplusia ni* larvae, topical application of thymol inhibited GST activity by 41% (Tak et al., 2017), whereas topical application of thymol on larvae of *Plutella xylostella* (Kumrungsee et al., 2014), and oral administration in *Ephestia kuehniella* (Shahriari et al., 2018) and *Tuta absoluta* (Piri et al., 2020) increased GST activity. At LC_50_ of thymol we obtained that there was no difference in GST activity between females and males. However, because initial activity in control females was higher, a higher percentage of inhibition was recorded in females than males (58 vs. 35%). Sexual dimorphism in detoxification enzyme activity has also been revealed in other studies where pest insects were exposed to plant-derived compounds. In *C. maculatus* GST, p-NPA esterase and α-esterase were not affected by lemongrass oil, whereas β-esterase was inhibited only in females (de Souza et al., 2019).

### Thymol Increases Activities of Phase I Detoxification Enzymes

Two enzymes involved in the phase I detoxification, CarE and MFO, increased the activity in response to thymol suggesting that they could have an important role in thymol metabolism in the bean weevil females and males. Further investigations are needed to elucidate how bean weevils detoxify thymol. In mammals, the majority of thymol is rapidly excreted unchanged or as a conjugate but oxidation of methyl and isopropyl groups also occurred (Austgulen et al., 1987). In difference to our results, thymol did not change the activity of CarE and MFO in *Trichoplusia ni* (Tak et al., 2017) which larvae excreted thymol bound to glucose without the change in its monoterpenoid structure (Passreiter et al., 2004). Highly diverse results have been obtained in other insect species where thymol had insignificant effect on esterase and MFO activities (Yotavong et al., 2015) or provoked their induction (Boncristiani et al., 2012; Kumrungsee et al., 2014; Piri et al., 2020) or inhibition (Waliwitiya et al., 2012; Shahriari et al., 2017; Gaire et al., 2021).

Comparison between thymol treated females and males revealed that males had higher CarE and MFO activity and provoked activity increase at lower thymol concentrations than females. It is not clear how detoxification enzyme activity variation contributes to the higher tolerance of females to insecticides. For example, females of *Helopeltis theivora* that are more tolerant to organophosphates had similar MFO activity to males and higher activity of α-esterase and GST (Roy and Prasad, 2018). Females of tortricid species *Lobesia botrana* and *Grapholita molesta* that are more tolerant to neonicotinoid insecticide thiacloprid and less tolerant to organophosphate insecticide chloropyrifos, exhibited sex-specific differences in detoxification enzymes (Navarro-Roldán et al., 2017, 2020). Namely, females of *L. botrana* had higher activities of MFO and GST and lower sensitivity of esterase to specific inhibitor DEF, whereas *G. molesta* females exhibited faster inhibition of MFO with specific inhibitor PBO. In *C. macullatus* treated with lemongrass oil females had higher p-NPA esterase activity (de Souza et al., 2019). Evidently, our results on lower activity of detoxification enzymes in more tolerant sex disagree with other studies on chemical and botanical insecticides. This may suggest an involvement of other mechanisms of tolerance such as better behavioral avoidance of a toxic compound or cuticle structure which slows-down thymol penetration (Panini et al., 2016). Besides, esterases and MFO are multifunctional enzymes encoded by a large number of genes organized into families (Feyereisen, 2012; Montella et al., 2012). Change in detoxification gene expression in response to xenobiotics is sex-biased and can be related to sex-specific differences in xenobiotic metabolism or other sex-specific physiological functions such as the production of pheromones and hormones by MFO and odorant degradation by esterases (Le Goff et al., 2006; Robert et al., 2013; Liu et al., 2019). High CarE activity that we recorded in male weevils might be related to its role in reproduction. It is known that carboxylesterases are overexpressed in the male reproductive tract of insects where they provide protection against xenobiotics and take part in sperm differentiation, maturation and function (Mikhailov and Torrado, 1999, 2000). In agreement with our results much higher esterase activity in males than females have been detected in whole body homogenates of *Lygus hesperus* (Zhu and Brindley, 1990) and abdomens of *Grapholita molesta* (de Lame et al., 2001) and *Cydia pomonella* (Fuentes-Contreras et al., 2007).

### Conclusion

In conclusion, we showed insecticidal activity of thymol against the bean weevil. Since thymol has been approved by the Environmental protection agency for use on food crops (EPA Code 080402, 2021) and has many human health promoting effects (de Alvarenga et al., 2021) it can be safely used as a contact insecticide on bean seeds. Results on bean weevil physiological responses have implications for designing of future thymol-based insecticides. Inhibition of AChE is responsible for fast mortality response. However, thymol-induced inhibition was weak and thus involvement of other neurotoxicity and/or metabolic targets cannot be excluded. Due to inhibition of GST thymol can be used to synergize effects of chemical insecticides for which GST activity is crucial and thus reduce applied doses of these dangerous compounds. Since thymol induce the activity of MFO and CarE future formulations of thymol-based insecticides should involve inhibitors of these enzymes. Sex-specific differences in tolerance to thymol and sex-specific physiological responses to thymol exposure (especially high CAT and CAT to SOD activity ratio in females, and high CarE activity in males) should be also taken into account in bean weevil management. Further studies are needed to fully elucidate mechanisms of thymol toxicity and tolerance.

## Data Availability Statement

The raw data supporting the conclusions of this article will be made available by the authors, without undue reservation.

## Author Contributions

JL, IK, and DŠJ designed the experiment. JL, DŠJ, and SJ conducted the experiment. AV and SMJ determined enzyme activities. DŠJ and MK performed statistical analysis. JL, DŠJ, and IK prepared the manuscript. All authors have read and agreed to the published version of the manuscript.

## Conflict of Interest

The authors declare that the research was conducted in the absence of any commercial or financial relationships that could be construed as a potential conflict of interest.

## Publisher’s Note

All claims expressed in this article are solely those of the authors and do not necessarily represent those of their affiliated organizations, or those of the publisher, the editors and the reviewers. Any product that may be evaluated in this article, or claim that may be made by its manufacturer, is not guaranteed or endorsed by the publisher.
